# Multi-Layer Sparse Representation for Weighted LBP-Patches Based Facial Expression Recognition

**DOI:** 10.3390/s150306719

**Published:** 2015-03-19

**Authors:** Qi Jia, Xinkai Gao, He Guo, Zhongxuan Luo, Yi Wang

**Affiliations:** School of Software, Dalian University of Technology, Dalian 116621, China; E-Mails: jiaqi7166@gmail.com (Q.J.); gaoxinkai@mail.dlut.edu.cn (X.G.); zxluo@dlut.edu.cn (Z.L.); wangyi_dlut@126.com (Y.W.)

**Keywords:** facial expression recognition, local binary patterns, weighted patches, sparse representation, multi-layer model

## Abstract

In this paper, a novel facial expression recognition method based on sparse representation is proposed. Most contemporary facial expression recognition systems suffer from limited ability to handle image nuisances such as low resolution and noise. Especially for low intensity expression, most of the existing training methods have quite low recognition rates. Motivated by sparse representation, the problem can be solved by finding sparse coefficients of the test image by the whole training set. Deriving an effective facial representation from original face images is a vital step for successful facial expression recognition. We evaluate facial representation based on weighted local binary patterns, and Fisher separation criterion is used to calculate the weighs of patches. A multi-layer sparse representation framework is proposed for multi-intensity facial expression recognition, especially for low-intensity expressions and noisy expressions in reality, which is a critical problem but seldom addressed in the existing works. To this end, several experiments based on low-resolution and multi-intensity expressions are carried out. Promising results on publicly available databases demonstrate the potential of the proposed approach.

## 1. Introduction

Expression is a basic way to express human’s emotion, and it is also an effective non-verbal communication method. Therefore, automatic facial expression recognition (AFER) has become more and more important and plays an important role in computer vision. Facial expression recognition has wide application prospects in areas such as human-computer interface (HCI), image retrieval and psychological research.

According to different facial expression features, existing facial expression analysis approaches can be categorized into static images-based methods and dynamic image sequences-based methods. Methods based on dynamic image sequences are often used to enhance recognition performance by use of longitudinal changes information in facial image sequences [1,2]. However, they require more facial images and more computation time to analyze the facial information, which are very challenging in many situations. Thus wide attention has been paid to static images-based methods [3], and our work is also based on this.

Static images-based methods mainly consist of two stages: feature extraction and classifier design. Deriving an effective facial representation from original face images is a vital step for successful facial expression recognition. Many efforts have been devoted to two classical methods: geometric-based methods and appearance-based methods [4,5,6,7]. Geometric-based methods analyze the relationship of facial components and extract facial features with shape and locations [8]. However, in reality it is difficult to detect and track the feature points of facial expressions accurately and fast. As a contrast, the features of appearance-based methods are extracted from the whole face, such as Gabor-wavelet feature, local binary pattern feature. Gabor-wavelet feature [9] is widely used to describe facial expression with a bank of Gabor filters. Zhang *et al.* [10] utilized the features of facial elements and muscle movements by extracting patch-based 3D Gabor features. However, it is time-consuming to calculate a mass of different Gabor coefficients. In [11] the input images are first subjected to local, multi-scale Gabor-filter operations, and then the resulting Gabor decompositions are encoded using radial grids, imitating the topographical map-structure of the human visual cortex. There are also some similar approaches that divide a face image into certain small blocks, and apply some feature extraction algorithms, such as local binary pattern (LBP) analysis and scale invariant feature transformation (SIFT) [12,13,14] in order to get a local texture description. The local binary pattern (LBP) feature [9,12] improves expression recognition performance and efficiency because of its tolerance of illumination changes and compactness. However, an important fact that has often been neglected is that some patches such as the eyes, mouth, and nose provide more important information in facial expression recognition [15]. In our work, an improved LBP descriptor with spatial feature information is used. The Fisher separation criterion is employed to automatically calculate corresponding weights for all the facial patches without supervision.

On the other hand, in the stage of classifier design, many classical methods have been commonly adopted, such as linear discriminant analysis (LDA), linear programming (LP) and support vector machines (SVM), *etc.* LDA is a subspace learning method which can represent facial deformations in lower feature space. LP recognizes facial expressions by simultaneous feature selection and classifier training. SVM is a popular and powerful classifier technique for many classification problems. SVM maps the feature vectors into higher dimensional feature space by appropriate kernels, and finds the maximal margin to separate different facial expressions. Shan *et al.* [16] compared such several classifier methods and concluded that SVM achieved better performance and was more robust for low resolution expression images. In [17], SVM is combined with a local Steerable Pyramid Transform (SPT) feature for facial expression verification. A newly developed method shows good discrimination ability by integrating curvelet transform and online sequential extreme learning machine with radial basis function (RBF) hidden nodes [18]. Sparse representation-based classification (SRC), which is derived from the compressive sensing (or compressive sampling—CS) theory [19,20], has been successfully used for face recognition [21]. Other research based on SRC have also shown promising results in the field of facial expression recognition during the past few years, and achieved better performances than many classical algorithms [22,23,24].

Although numerous methods have been proposed over the years, facial expression recognition still remains a significant challenge to recognize different human expression styles as well as their large inter-personal variations. In order to improve recognition rates in reality, many factors that complicate facial expression analysis should be taken into account, such as low-resolution, low-intensity and noisy facial expressions. Many efforts have been paid to solve low-resolution conditions. Tian [4] performed experiments on low resolution with geometric features and Gabor features, and a three-layer neural network was used as classifier. Then, Shan *et al.* [16] improved the recognition rate with LBP feature, instead of geometric and Gabor features, which are difficult to detect and track facial components in low-resolution images, and LBP features are more robust over a range of low resolutions. Moreover, Shan’s work also showed that compared to a neural network, SVM could provide better performance with the same expression features. On the other hand, most works choose extreme facial images with high intensity from the whole sequences to construct the experimental database. However, there are usually many different expression intensities that human beings can express in reality. Some low intensity expressions are very similar, such as fear and sadness, which always have low recognition rates in most existing methods. For this problem, Ekman [25] developed the Facial Action Coding System (FACS), which is used to identify facial expressions called micro-expressions. It usually occurs in high-state situations which are very brief in duration, lasting only 1/25 to 1/15 of a second. This system measures the relaxation or contraction of each individual muscle and assigns a facial action unit. Thus, FACS must train and analyze a mass of facial expression images with supervision, and it is very difficult to perceive the action units of low-intensity expressions. Most of the systems developed for AFER cater only to controlled laboratory circumstances in which much of the expression performing is rehearsed and performed by trained actors. What is not currently known is how well these systems adapt to suboptimal circumstances in which there is image noise and occlusion in the images. Some works solve the problem by various filter methods, which is a usual way to handle noisy images [26]. Ouyang *et al.* [27] used Histograms of Oriented Gradient (HOG) descriptors and LBP conjunction with SRC separately to get two judgment vectors, and managed to fuse them to achieve a better performance. In this paper, we focus on the ability of the classifiers to handle noise.

In our former work, we formulated the static facial expression recognition problem by a sparse representation (SR) method, which casts the recognition problem as finding a sparse approximation of the test image in terms of the whole training set. SR can simultaneously reduce feature dimensions and classify expressions without training the classifier preliminarily [28]. In this paper, we utilize sparse representation for facial expression recognition, in which the training facial expression images are used as the dictionary to code an input testing facial image as a sparse linear combination of them via $l_{1}$-norm minimization. Intuitively, the desired representation for the testing facial expression is sparse, which should only be represented in terms of training instances of the same expression.

In this paper, we focus on the performance of SR on low resolution or noisy facial images. Moreover, considering the situation of multi-intensity based on the static facial expression, the implement of a multi-layer sparse representation (MLSR) model is elaborated, which is used to improve the recognition performance of multi-intensity facial expressions, especially for low intensity expression. More importantly, MLSR can be used in many similar classification problems. Experiments results show the robustness of our methods on low-resolution, multi-intensity and noisy facial expressions. Further, we also discuss the across-dataset which is a common problem in recognition tasks.

The paper is organized as follows: Local Binary Patterns are introduced in Section 2. Section 3 proposes the sparse representation method for facial expression recognition. Multi-layer sparse representation method is presented in Section 4. Experiments on facial expression recognition are listed in Section 5. Section 6 concludes this paper. 

## 2. Expression Feature Extraction

### 2.1. Local Binary Patterns (LBP) 

In this part, facial image is divided into several patches based on LBP features. LBP is a novel low-cost image descriptor for texture classification which can present salient micro-patterns of facial images. The original LBP operator was introduced by Ojala *et al.* [29], in which the image pixel is labeled as a binary class by setting a threshold for the difference between the center pixel and its neighbors as shown in Equation (1):

(1) $$LBP=\sum_{j=0}^{7}T(g_{j}-g_{c})\cdot 2^{j},T(x)=\{\begin{array}{c} 1,x\geq 0\\ 0,x<0 \end{array}$$

**Figure 1:**
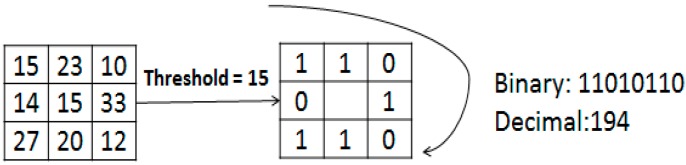
The basic LBP operator.

The concatenation of the neighboring labels is then used as a unique descriptor for each pattern, and its decimal number is the LBP feature of the center point. Figure 1 gives a simple example. In our experiments, the size of each patch is 20 × 20 pixels. The limitation of the basic LBP operator is that the 3 × 3 patch is too small to capture dominant features with large scale structures. Hence the operator is extended with different sizes [30]. Using circular neighborhoods and interpolating the pixel values allow any radius and number of pixels in the patch. Further extension of LBP is to use uniform patterns [31]. LBP is called uniform if it contains at most two bitwise transitions from 0 to 1 or *vice versa* when the binary string is considered circular. For example, 00110000 and 11100001 are uniform patterns. It is observed that uniform patterns account for nearly 90% of all patterns in the (8,1) patch and for about 70% in the (16,2) patch in texture images.

Here we adopt a LBP operator $LBP_{P,R}^{u2}$, where (P,R) denotes P sampling points on a circle of radius of *R*. The subscript represents using the operator in a (P,R) neighborhood; the superscript u2 indicates using only uniform patterns and labeling all remaining patterns with a single label.

### 2.2. Weighted Patches 

The histogram contains information about the distribution of the local micro-patterns, such as edges, spots and flat areas over the whole image. Thus a histogram of a labeled image p(x,y) can be defined as follows:

(2) $$H_{i}=\sum_{x,y}S(p(x,y)=i),i=0,\ldots ,n-1,\;\;(x,y)\in g(x,y)$$

where n is the number of different labels produced by the LBP operator. S(T)=1 when T is true and S(T)=0 when T is false.

Further, for efficient facial representation, feature extracted should also retain spatial information. Hence the facial image is divided into m small patches $R_{0},R_{1},\ldots ,R_{m}$ and a spatially enhanced histogram is defined as:

(3) $$H_{i}=\sum_{x,y}S(p(x,y)=i),\;i=0,\ldots ,n-1,\;(x,y)\in R_{j}$$

**Figure 2:**
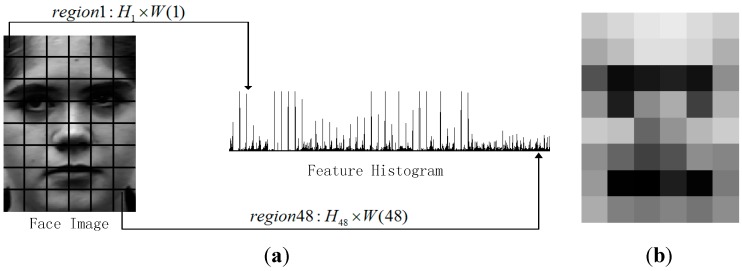
LBP feature extracting with weighted patches. (**a**) Histogram of LBP features; (**b**) Weighted patches.

Hence for each patch, $LBP_{8,2}^{u^{2}}$ operator will produce a 59 dimension descriptor. As shown in Figure 2a, a histogram with 59 labels is adopted to accumulate the value on each dimension for all the patches. For example, for a facial image with 120 × 160 pixels, there are 48 (6 × 8) patches (20 × 20 pixels for each patch). Then, the feature dimension of each facial image is 2832 (59 × 48). To exclude redundancy data, the feature data is projected into a PCA subspace. In the dimension reduction step 98% information is kept according to the reconstruction error.

Moreover, it is expected that some face patches (e.g., eyes, mouth, and nose) provide more important information than others in facial expression recognition. Therefore, different facial patches should have different weights. Shan *et al.* [12] designed the weights empirically and gave higher weights for interesting facial patches manually, which is not automatic and reasonable. In order to learn suitable weights for each patch automatically, an approach based on fisher separation criterion [32] is used to calculate weighs as follows.

We set C to be the number of facial expression classes. Let the similarities of different samples of the same facial expression compose the intra-expression similarity class, and samples from different facial expressions compose the extra-expression similarity class. For patch $R_{j}$ of the image, the mean value $M_{I}(j)$ and the variance $S_{I}^{2}(j)$ of the intra-expression similarity class can be computed as follows:

(4) $$M_{I}(j)=\frac{1}{C}\sum_{m=1}^{C}\frac{2}{N_{m}(N_{m}-1)}\sum_{k=2}^{N_{m}}\sum_{n=1}^{k-1}\Psi (H_{j}^{\left(m,n\right)},H_{j}^{\left(m,k\right)})$$

(5) $$S_{I}^{2}(j)=\sum_{m=1}^{C}\sum_{k=2}^{N_{m}}\sum_{n=1}^{k-1}{\left(\Psi (H_{j}^{\left(m,n\right)},H_{j}^{\left(m,k\right)})-M_{I}(j)\right)}^{2}$$

Similarly, the mean value $M_{E}(j)$ and the variance $S_{E}^{2}(j)$ of the extra-expression similarity class can be calculated as follows:

(6) $$M_{E}(j)=\frac{2}{C(C-1)}\sum_{m=1}^{C-1}\sum_{n=m+1}^{C}\frac{1}{N_{m}N_{n}}\sum_{k=1}^{N_{m}}\sum_{l=1}^{N_{n}}\Psi (H_{j}^{\left(m,k\right)},H_{j}^{\left(n,l\right)})$$

(7) $$S_{E}^{2}(j)=\sum_{m=1}^{C-1}\sum_{n=m+1}^{C}\sum_{k=1}^{N_{m}}\sum_{l=1}^{N_{n}}{\left(\Psi (H_{j}^{\left(m,k\right)},H_{j}^{\left(n,l\right)})-M_{E}(j)\right)}^{2}$$

where $H_{j}^{\left(m,n\right)}$ denotes the histogram extracted from $R_{j}$ region of the $n^{th}$ sample in $m^{th}$ class. $N_{m}$ is the sample number in $m^{th}$ class in the dataset. Here, we use the histogram intersection $\Psi (H^{1},H^{2})$ as the similarity measurement of two histograms. $\Psi (H^{1},H^{2})=\sum_{n=1}^{L}\min(h_{n}^{1},h_{n}^{2})$, Where $H^{1}$ and $H^{2}$ are two histograms, and L is the number of bins in the histogram.

Finally, the weight W(j) for $R_{j}$ is computed as Equation (8), and the weighted feature extracted from $R_{j}$ region of the $n^{th}$ sample in the $m^{th}$ class is $HW_{j}^{\left(m,n\right)}$. Figure 2b shows the final expression feature with weighted patches. The patches are expressed in gray level from 0 to 255. The darker the patch is, the larger the weight is. It is obvious that the weight is set reasonably:

(8) $$W(j)=\frac{{\left(M_{I}(j)-M_{E}(j)\right)}^{2}}{S_{I}^{2}(j)+S_{E}^{2}(j)},HW_{j}^{\left(m,n\right)}=W(j)\times H_{j}^{\left(m,n\right)}$$

## 3. Facial Expression Recognition Based on Sparse Representation

The SR based classification (SRC) for facial expression is conducted by evaluating which class of training samples can achieve the minimum reconstruction error with the input testing image by the sparse coding coefficients. Suppose we have C expression classes, and let $A=[A_{1},A_{2},\ldots ,A_{C}]$ be the concatenation of the n training samples from all the C classes, where $n=n_{1}+n_{2}+\ldots +n_{C}$, then the linear representation of $y_{0}$ can be written in terms of all training samples as $y_{0}=A\alpha$.

However, usually, the feature vector of an image is very high-dimensional and the number of training samples is limited, so A is over-determined. The least-squares solution can exhibit severe bias for “the curse of dimensionality” caused by over-determined. Luckily, the CS theory has shown that a sparse signal can be recovered from a small number of its linear measurements with high probability [20]. According to CS, a sparse signal $x\in R^{n}$ should be recovered from the following linear random projections: y=Φx, where $y\in R^{m}$ is the measurement vector, $\Phi \in R^{m\times n}(m<<n)$ is a random projection matrix. For facial expression features, the projection from the image space to the feature space can be represented as a measure matrix Φ. Therefore, the measure vector is $y=\Phi y_{0}=\Phi A\alpha$.

**Figure 3:**
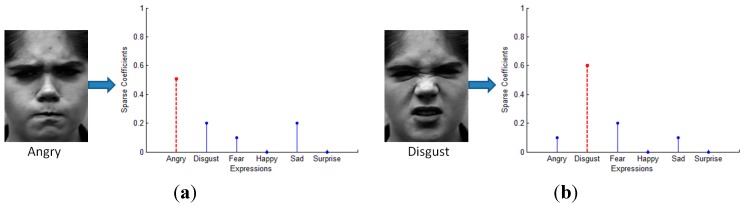

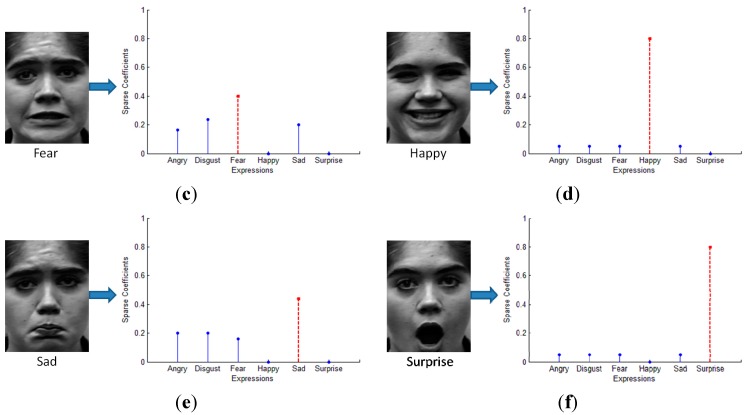
Classification result for different testing expressions (**a**) Angry; (**b**) Disgust; (**c**) Fear; (**d**) Happy; (**e**) Sad; (**f**) Surprise.

Considering the existence of error, the formulation can be rewritten as $y=\Phi A\alpha +e_{0}=\bar{A}\alpha +e_{0}$, where $e_{0}$ is a noise term with bounded energy ${\left\|e_{0}\right\|}_{2}<\varepsilon$ and $\bar{A}=\Phi A$. Then our goal is to solve the convex optimization problem: compute $\min{\left\|\alpha \right\|}_{1}$ subject to ${\left\|y-\bar{A}\alpha \right\|}_{2}\leq \varepsilon$. The sparse representation based facial expression recognition algorithm is summarized in Algorithm 1. The result is shown in Figure 3. As we can see, there are six basic emotions (anger, disgust, fear, happy, sad, surprise), and the test image shows obvious difference to the other expressions, especially in Figure 3d,f. Meanwhile, the other four expressions are a little bit similar in some instance, as shown in Figure 3a–c,e. In most existing methods, the result will get worse when the test image is low-intensity facial expression. However, the SR based classification method shows stable recognition rate, which will be demonstrated in the next Section and evaluated in the experiments.

**Algorithm 1.** Facial Expression Recognition based on Sparse Representation**Input:** a matrix of training images $A\in R^{d\times n}$ for *C* expression classes, a linear feature transform $\Phi \in R^{m\times d}$, a test image $y_{0}\in R^{n}$, and an error tolerance ε.
**Output:** identity (*y*) = argmin $r_{i}(y)$.
**1.** Compute $y=\Phi y_{0}$ and $\bar{A}=\Phi A$, and normalize y and columns of $\bar{A}$ to unit length.**2.** Solve the following convex optimization problem
$\min{\left\|\alpha \right\|}_{1}\;\text{subject}\;\text{to}\;\|{y-\bar{A}\alpha \|}_{2}\leq \varepsilon$**3.** Compute the residuals:
$$r_{i}(y)={\left\|y-\bar{A}\delta_{i}(\alpha )\right\|}_{2},\;\text{for}\;i=1,2,\ldots ,C$$
where $\delta_{i}(\cdot ):R^{n}\to R^{n}$ is the characteristic function which selects the coefficients associated with the $i^{th}$ class.

## 4. Multi-Intensity Facial Expression Recognition Based on Multi-Layer Sparse Representation

**Figure 4:**
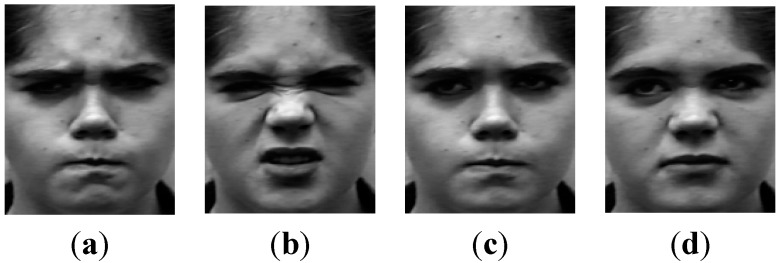
Anger and Disgust in different intensity (**a**) Anger in high intensity; (**b**) Disgust in high intensity; (**c**) Anger in low intensity; (**d**) Disgust in low intensity.

Most existing research in facial expression analysis mainly focuses on recognizing extreme facial expressions. However, in real-world applications, such as human-computer interaction and data-driven animation, low-intensity or subtle facial expressions are more universal than high-intensity or exaggerated expressions. Some low-intensity facial expressions are very similar, such as anger and disgust, which always have low recognition rate in most existing approaches. The essential reason is that these methods neglect the correlations among different intensities. As shown in Figure 4, Figure 4a,c are “anger”, while Figure 4b,d are “disgust”. In the high intensity version, Figure 4a,b have obvious differences at the nose. However, in the low intensity form, Figure 4c,d are nearly the same. In a traditional classifier, Figure 4b,d are trained in the same class, in which the details of low intensity expression may be weakened by high intensity images.

**Figure 5:**
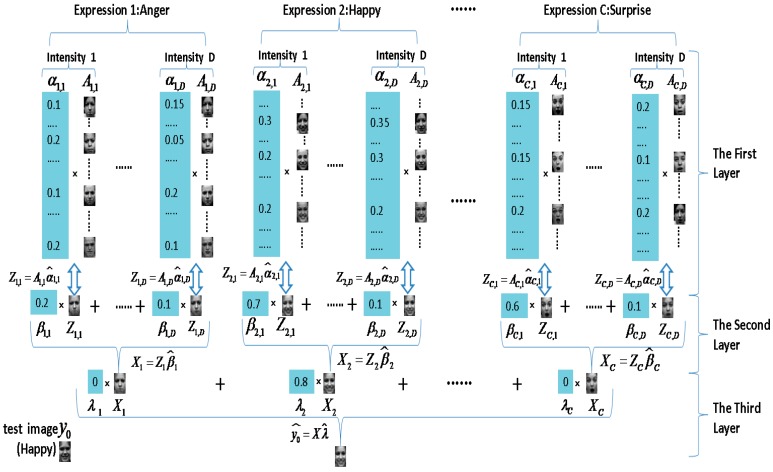
Multi-layer sparse representation model.

Driven by this purpose, a multi-layer sparse representation (MLSR) method is proposed with more detailed categories to improve the recognition performance of multi-intensity facial expressions. Our notion is motivated by the latest work of Liu *et al.* [30], which casts sparse representation as nonnegative curds and whey (NNCW). The MLSR model consists three layers: the first layer is used to calculate sparse correlations among each expression intensity of each expression class; the second layer codes the testing expression with all intensity groups of each expression class; the third layer is set to represent the testing facial expression among different expressions. Therefore, the MLSR technique can present the correlations within a same intensity and the disparities between different intensities, which are beneficial to the situation of multi-intensity facial expressions. As shown in Figure 5, on the first layer of MLSR, each expression is divided into *D* different intensity. All the images with the same expression and the same intensity are trained together, which means images with the same expression and different intensity are trained separately. Therefore, an easily confused low intensity expression has stronger correlations with the corresponding intensity class. 

Considering the correlations are also strong in the same expression of different intensity. On the second layer, we combine different intensity of each expression separately to strengthen the correlations. Finally, the last layer gives the classification results.

The entire MLSR method is summarized in Algorithm 2. We assume that there are C facial expression classes and each expression class has D types of intensity (Note each facial expression has the same D here). The images set of the $j^{th}$ type of intensity of the $i^{th}$-class expression is defined as $A_{i,j}$, and $N_{i,j}$ is the image number in $A_{i,j}$.

On the first layer, linear regression models of B(B=C×D) are considered by treating $y_{0}$ as the response, and each image from $A_{i,j}$ has one basis: $y_{0}=A_{i,j}\alpha_{i,j}$. That is, the regression problem of the $j^{th}$ type of intensity of the $i^{th}$-class expression is $y=\Phi_{i,j}y_{0}+e_{0}^{i,j}=\Phi_{i,j}A_{i,j}\alpha_{i,j}+e_{0}^{i,j}={\bar{A}}_{i,j}\alpha_{i,j}+e_{0}^{i,j}$, where i=1,…,C, j=1,…,D, $\alpha_{i,j}\in R^{N_{i,j}\times 1}$ as denoted in Section 3. The result leads to B sparse representations of y, which can be express as $Z_{i,j}=A_{i,j}{\overset{˰}{\alpha }}_{i,j}$ and ${\overset{˰}{\alpha }}_{i,j}$ is reconstruction coefficients learned from samples within the $j^{th}$ type of intensity of the $i^{th}$-class expression.

On the second layer, C linear regression models are built. The representation of $y_{0}$ is refined by the representations of $y_{0}$ obtained in the first stage: $y_{0}=Z_{i}\beta_{i},Z_{i}=[Z_{i,1},\ldots ,Z_{i,D}],\beta_{i}\in R^{D\times 1}$. That is, the $i^{th}$ regression problem is based on: $y=\Phi_{i}y_{0}+e_{0}^{i}=\Phi_{i}Z_{i}\beta_{i}+e_{0}^{i}={\bar{Z}}_{i}\beta_{i}+e_{0}^{i}$. The result of the second layer leads to C sparse representations of y, which can be expressed as $X_{i}=Z_{i}{\overset{˰}{\beta }}_{i}$. ${\overset{˰}{\beta }}_{i}$ is reconstruction coefficients learned from samples within the first layer’s representations for the $i^{th}$-class expression.

**Algorithm 2.** Multi-intensity Facial Expression Recognition based on Multi-Layer Sparse Representation**Input:** training images $\left[A_{1,1},\ldots ,A_{1,D},\ldots ,A_{C,1},\ldots ,A_{C,D}\right]$; measure matrixes of each layer $\left[\Phi_{1,1},\ldots ,\Phi_{1,D},\ldots ,\Phi_{C,1},\ldots ,\Phi_{C,D}\right]$, $\left[\Phi_{1},\ldots ,\Phi_{C}\right]$, Φ;
A test image $y_{0}\in R^{n}$, and error tolerance ε.
**Output:** identity (*y*) = argmin $r_{i}(y)$.
**1. The First Layer:** Solve the following convex optimization problem in each intensity of each expression class.
$\min{\left\|\alpha_{i,j}\right\|}_{1}\;\text{subject}\;\text{to}\;{\left\|y-{\bar{A}}_{i,j}\alpha_{i,j}\right\|}_{2}\leq \varepsilon \;\text{for}\;i=1,\ldots ,C,j=1,\ldots ,D$**2. The Second Layer:** Solve the the testing facial expression and all intensity groups in the same expression. following convex optimization problem between
$\min{\left\|\beta_{i}\right\|}_{1}\;\text{subject}\;\text{to}\;{\left\|y-{\bar{Z}}_{i}\beta_{i}\right\|}_{2}\leq \varepsilon \;\text{for}\;i=1,\ldots ,C$**3. The Third Layer:** Solve the following convex optimization problem of the testing facial expression among different expressions
$$\min{\left\|\lambda \right\|}_{1}\;\text{subject}\;\text{to}\;{\left\|y-\bar{X}\lambda \right\|}_{2}\leq \varepsilon$$
**4.Compute the residuals:**
$$r_{i}(y)={\left\|y-{\overset{˰}{\lambda }}_{i}{\bar{X}}_{i}\right\|}_{2}\;\text{for}\;i=1,\ldots ,C$$

Finally, the third layer further refines the representation of $y_{0}$ by using the representation of $y_{0}$ obtained in the second stage: $y_{0}=X\lambda ,X=[X_{1},\ldots ,X_{C}],\lambda =[\lambda_{1},\ldots ,\lambda_{C}]\in R^{C\times 1}$.

As we can see, the final regression problem is:

(9) $$y=\Phi y_{0}+e_{0}=\Phi X\lambda +e_{0}=\bar{X}\lambda +e_{0}$$

Then, the Dynamic Group Sparsity (DGS) [33] algorithm is used to solve the convex optimization problem, which is more accurate and faster than conventional lasso methods.

**Figure 6:**
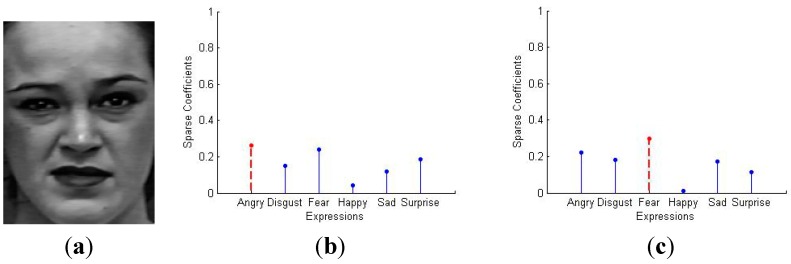
Recognition result of “Fear” with SR and MLSR. (**a**) “Fear” in low intensity; (**b**) Recognition result with SR; (**c**) Recognition result with MLSR.

In order to explain the roles of each layer in detail, a low intensity expression “Fear” is selected as shown in Figure 6a. In the SR classifier, it is recognized as “Anger” as shown in Figure 6b. However, it exhibits obvious discrimination with other expressions with MLSR, as shown in Figure 6c.

## 5. Experiments

### 5.1. Data Set 

The Cohn-Kanade Dataset (CK) [34], Extended Cohn-Kanade Dataset (CK+) [35] and the JAFFE Dataset [36] are used in our experiments, which are the most comprehensive datasets for facial expression recognition. The CK+ Dataset’s participants were instructed by an experimenter to perform a series of 23 facial displays. Each display began in a neutral face. Image sequences were digitized into either 640 × 490 or 640 × 480 pixel arrays with 8-bits gray-scale or 24-bit color values. On the other hand, the JAFFE Dataset contains 213 images of seven facial expressions (six basic facial expressions and one neutral expression) posed by 10 Japanese female models. Ten expressers posed three or four examples of each expression.

First of all, face images are normalized to a fixed distance between the centers of two eyes. The fixed distance is 60 pixels. It is observed that the width of a face is roughly two times of the distance, and the height is roughly three times. Hence, facial images of 120 × 160 pixels are cropped from original frames based on the two eyes location. Some parameters can be optimized for the LBP feature selection. The first one is the LBP operator, and another is the number of patches divided, which has been illuminated in Section 2.

### 5.2. Sparse Representation for 7-Class Facial Expressions Recognition

In our experiments, 309 image sequences are selected from the CK+ Database. The only selection criterion is that a sequence can be labeled as one of the six basic emotions (angry, disgust, fear, happy, sad, surprise). The database is presented the inventory of the six basic expressions as Table 1. For each sequence, the neutral face and three peak frames are used for prototypic expression recognition, resulting in 1236 images (135 Angry, 177 Disgust, 75 Fear, 207 Happy, 84 Sad, 249 Surprise and 309 Neutral). The sequences come from 106 subjects. To evaluate the generalization performance to novel subjects, a 10-fold cross validation testing scheme is adopted as [12]. However, our results have no comparability to Shan’s work because of different database, experimental setups, pre-processing procedures, weighted patches, and classifier. But we can also draw many conclusions from the following experiments.

First, the performance of weighted patches which is introduced in Section 2 is analyzed. As shown in Table 2, the recognition results based on SR method with weighted patches is 87%, which performs better than the method without weights of 85.1%. On the other hand, to verify the effectiveness of sparse representation for facial expression recognition, SVM is used as the classifier for comparison, which has been a popular technique for facial expression recognition. Here we use the SVM implementation with linear kernel and RBF kernel in the public available machine learning library SPIDER [37], and both methods are carried out with weighted patches proposed in our paper. Moreover, in order to highlight the classification performance of our methods (SR and MLSR), the following experiments about SVM are all performed with weighted patches which is to keep the same input for comparison. In sparse representation’s implementation, DGS algorithm [33] is used to solve the convex optimization problem. The comparison in Table 2 illustrates that sparse representation method outperforms SVM under the same experimental conditions.

**Table 1:** Number of each basic expression’s sequences.

Emotion	Numbers
Angry	45
Disgust	59
Fear	25
Happy	69
Sadness	28
Surprise	83
Total	309

**Table 2:** Comparisons between SR and SVM.

Methods	Recognition Results
Sparse Representation (patches without weights)	85.1%
Sparse Representation (patches with weights)	87%
SVM with linear kernel (patches with weights)	85.4%
SVM with RBF kernel (patches with weights)	86.5%

### 5.3. Data Set Performance over Different Resolutions

In many facial expression recognition applications, the input facial images are often low-resolution. We further evaluated the SR-based algorithm over a range of image resolutions, investigating its performance against low-resolution images. Similarly, SVM (linear kernel) is used for comparison. The lower resolution images are down-sampled from the original images. To get enough features from the lower resolution images, lower resolution images are resized to the size of the original images (120 × 160) by simple interpolation methods and then extract expression feature as above. 

**Figure 7:**
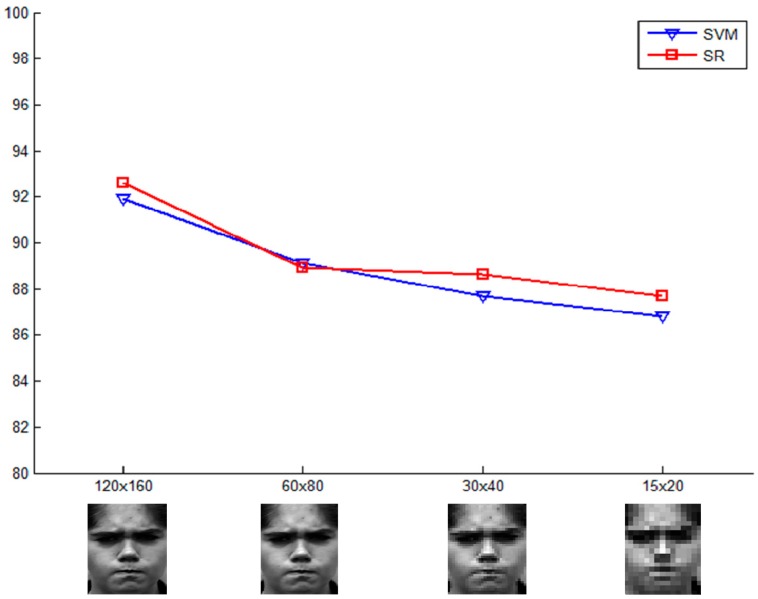
Comparisons on image resolutions between SR and SVM.

The recognition rates of 6-class expressions (without neutral expression) are shown in Figure 7. It is observed that the SR method is more effective for the great mass of resolutions than the SVM method. It indicates that sparse representation is robust for low-resolution expression images as SVM method. Besides, the recognition rate for the original image is 92.6%, which is higher than the result in Table 2, for the former data set is 6-class and the latter is 7-class.

### 5.4. MLSR for Multi-Intensity Facial Expression Recognition

#### 5.4.1. Recognition Results for Different Intensity

**Figure 8:**
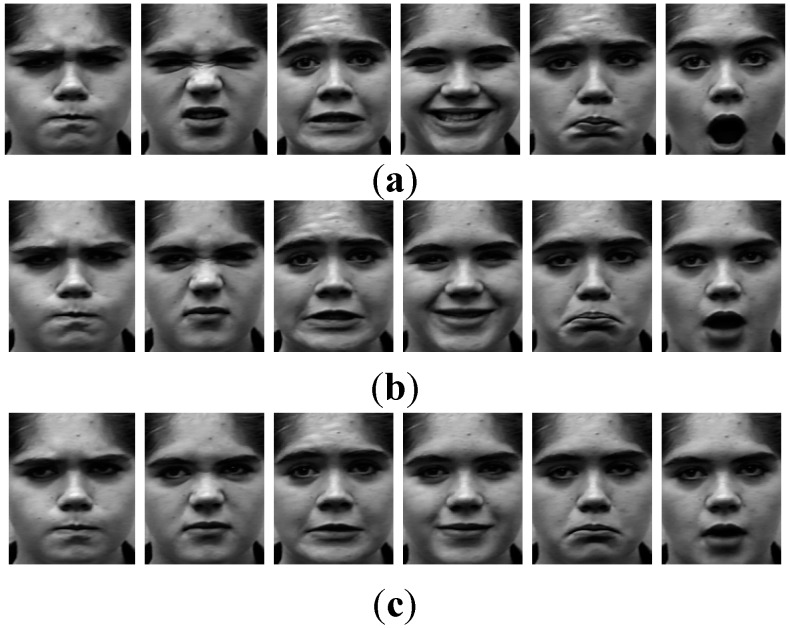
Multi-intensity example of six expressions: Angry, Disgust, Fear, Happy, Sad, Surprise. (**a**) High intensity; (**b**) Moderate intensity; (**c**) Low intensity.

**Figure 9:**
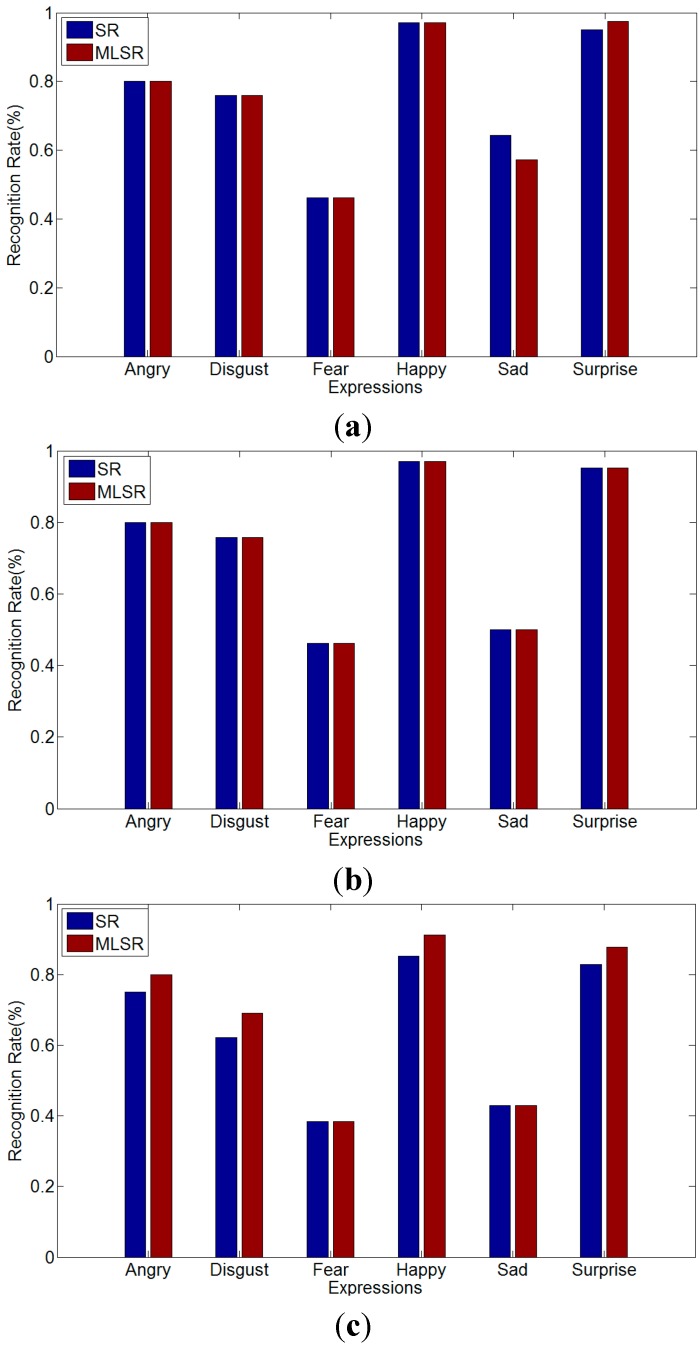
The comparison between MLSR and SR about multi-intensity recognition. (**a**) High intensity; (**b**) Moderate intensity; (**c**) Low intensity.

In this experiment, three frames are chosen from each expression sequence, as shown in Table 1. The chosen three frames have different expression intensity: one is high intensity which is the last frame in the sequence, one is moderate intensity which is selected in the middle of the sequence, and one is low intensity which is in the front half of the sequence. Therefore, our experiment consists of 6-class expressions and each expression includes three types of expression intensity. In Figure 8, there is an example of multi-intensity expressions. Half of all the sequences are used for training, and the rest half are used to test. Therefore, the testing is also person-independent. To verify the performance of MLSR, SR and SVM are used for comparing, which are presented in Figure 9 and Table 3.

As we can see in Figure 8, the testing data are divided into three parts (high intensity, moderate intensity, low intensity), and the recognition rate is calculated separately. For high intensity and moderate intensity, MLSR do not get better result than SR, but MLSR presents better accuracy for low-intensity expressions. However, in Figure 9c, the fear and sad expression show no differences like the other expressions for MLSR and SR. This is because the low-intensity sequences of fear and sad expressions are much more alike and difficult to distinguish, even for human beings. It is predictable that MLSR would achieve some improvements for high intensity and moderate intensity, if the database is more plentiful. Table 3 shows the results of multi-intensity expression recognition between MLSR and SVM. Likewise, MLSR and SVM are similar for high intensity and moderate intensity. However, for low intensity, MLSR is better than SVM.

**Table 3:** Comparisons on intensities between MLSR and SVM.

Methods	High Intensity	Moderate Intensity	Low Intensity
MLSR	83.56%	82.26%	76.35%
SVM	84.07%	81.43%	71.96%

#### 5.4.2. Comparison with State-of-The-Art Performance

In order to follow the same protocol, The Cohn-Kanade (CK) Dataset [34] is introduced to make a comparison between MLSR and state-of-the-art approaches. And the person-independent strategy is used for cross-validation. 

The CK database contains facial expression images of 97 university students (35% males and 65% females. 15% African–American and 3% Asian or Latino). Each person has a set of image sequences from neutral to certain facial displays coded by action units. For our study, we first select those sequences (from 92 persons) that can be labeled using one of the six basic expressions. For every person, we select three images for each expression the same as in the CK+ database (one is high intensity which is the last frame in the sequence, one is moderate intensity which is selected in the middle of the sequence, and one is low intensity which is in the front half of the sequence). Therefore, we select 296 sequences and there are 296 × 3 = 888 images in total for our experiments. All the images are separated into 10 sets, and all images of one subject are included in the same set. To evaluate the generalization performance to novel subjects, a 10-fold cross validation testing scheme is adopted.

Note that the results of different algorithms may not be directly comparable because of differences in experimental setups, the number of subjects and so on, but they can still indicate the discriminative performance of each approach.

**Table 4:** Comparison with state-of-the-art performance of the proposed algorithm on the CK database.

Methods	Subjects	Measure	Recognition Rate
[6]	97	5-fold	90.90%
[7]	90	-	93.66%
[10]	92	leave-one-subject-out	94.48%
[13]	97	10-fold	96.26%
[14]	90	leave-one-subject-out	96.33%
[12]	96	10-fold	88.4% (92.1%)
[11]	94	10-fold	91.51%
[18]	92	leave-one-subject-out	95.17%
MLSR	92	10-fold	92.3%

Table 4 shows a similar comparison with respect to the CK database. The first five methods belong to a dynamic approach which based on video sequences, and the last four approaches, including ours, belong to a static approach which is normally applied to static images. The dynamic approach uses the relations between the images in the videos, which needs more information. Most methods give better results than the static one, and [14] got the best recognition accuracy of 96.33%. However, in real-world applications, such as human-computer interaction and data-driven animation, static images are more universal than videos for expression recognition. Among the static approaches, our recognition accuracy is 92.3% which is just lower than [18]. In [18] a curvelet transform is used to get the features of the expression, and it is more sensitive to noise. However, our approach can remain robust over noise images, which is shown in the next Section.

### 5.5. Performance over Noise

**Figure 10:**
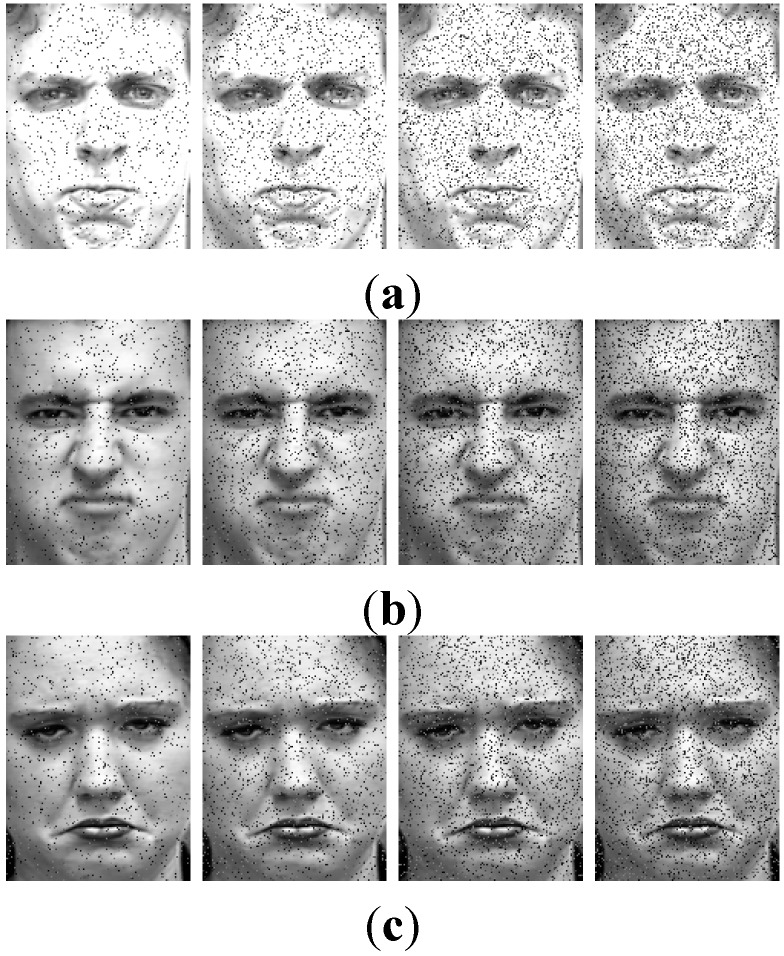
Facial expression with noise. (**a**) Anger; (**b**) Disgust; (**c**) Sadness.

In order to illustrate the robustness of SR over noise, Gaussian noise is imposed on the database with the standard deviation varying from 5 to 20. Some examples are shown in Figure 10. Figure 10a is the expression of anger corrupted by the noise whose standard deviations are 5, 10, 15, 20, respectively. Figure 10b,c are expressions of disgust and sadness, accordingly. The expressions are blurred when the noise is severe.

The experiments are performed on 7-class facial expressions, and the recognition rates are shown in Table 5. The recognition results of each expression class are illustrated in each column. For each expression class, the recognition rates drop gradually as the noise are heaver. There is a sensible difference between different expression classes. For some distinguishable expression such as disgust, happy and surprise, the recognition rates are as high as 84%–95%. Even when the noise is severe at 20%, the recognition rates are around 84%–91%. However, the recognition rates of some undistinguishable expressions are lower relatively, which is around 50%–77%. The average recognition rates over each noise degree are shown in the last column. The recognition rate of each expression class multiplied by the percent of its number over the whole database gives the results, which are around 77%–84%. The results show that SR can remain robust against noise in different degrees.

**Table 5:** Recognition rate of SR over noisy images.

	Expression	Angry	Disgust	Fear	Happy	Neutral	Sad	Surprise	Average
Noise									
5		77.048	92.325	68.055	94.662	78.125	56.945	92.870	83.712
10		71.238	89.231	67.499	93.015	77.438	53.055	91.144	81.559
15		67.370	85.845	61.945	92.301	75.915	50.056	89.147	79.205
20		61.946	83.952	56.111	91.190	73.364	49.168	87.897	76.856

**Figure 11:**
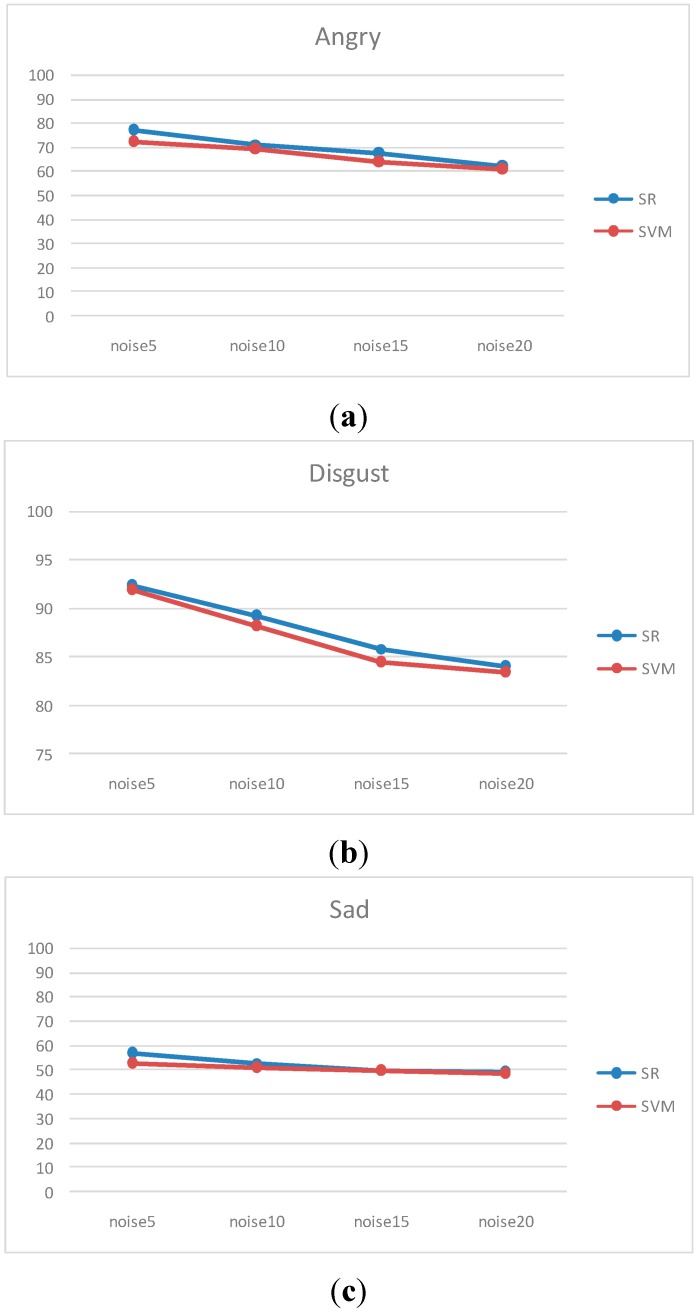
The comparison between SR and SVM against noise. (**a**) Angry; (**b**) Disgust; (**c**) Sad.

We also make a comparison with SVM. The performance is nearly equivalent to SR. However, for some expressions such as angry, disgust and sad, SR can achieve better performance than SVM. The result is shown in Figure 11. The recognition rate of both methods drops gradually as the noise increases. SR steadily performs better than SVM when the noise power increases.

### 5.6. Cross-Database Experiment

In general, most researchers do experiments using a single dataset for training and testing. That is, part of the dataset is employed for training the classifier, and the rest is used for testing the classifier. However, in the real world, we can’t ensure that the test dataset comes from the same dataset as the training dataset. Therefore, to verify a classifier’s robustness between different datasets, across-dataset experiments become very essential. Shan *et al.* [12] performed across-dataset experiments which trained on the CK database and due to the different controlled environments, such as atmospheres, image equipment and illumination. Therefore, the current expression classifier which is trained on a controlled environment’s database can only work well for a testing database with the same controlled environment. In this paper, we choose the Extended Cohn-Kanade Dataset as training database, and the JAFFE Dataset as testing database. Likewise, we take SR and SVM for comparisons, as shown in Table 6. Both SR and SVM methods achieve very low recognitions which are even below 50%.

**Table 6:** Comparisons on across-database between SR and SVM.

Methods	SR	SVM
Train: CK+ Test: JAFFE	40.5%	39.4%

**Figure 12:**
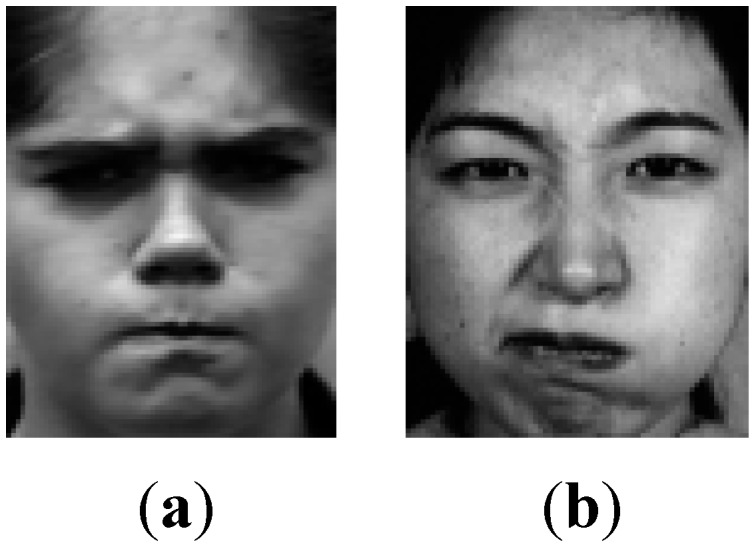
The same expression from different datasets. (**a**) The CK+ dataset; (**b**) The JAFFE dataset.

Further, the lack of standard evaluation criteria of basic expressions is also a crucial reason. Different expression databases have different evaluation criteria. They are consistent in general, but there are variances which influence the recognition. Figure 12 shows an example from the CK+ and JAFFE Datasets, in which the same angry expressions come from different databases. The left one is from the CK+ database, and the right one is from the JAFFE database. There are obvious differences between the two images, such as the eyebrows, mouth, and cheek. Therefore, the problem of how to establish a standard evaluation criterion for basic expressions remains to be resolved.

## 6. Conclusions

In this paper, a novel facial expression recognition based on sparse representation is proposed. First of all, the Fisher separation criterion is introduced to calculate the weight of LBP patches, which enhances the effect of important regions, such as the mouth and eyes. The most important contribution of this paper is the introduction of sparse representation as a facial expression recognition method. We have compared the proposed method with SVM, and low-resolution and noisy facial images are also taken into account. Furthermore, considering multi-intensity facial expression in the real-world, a multi-layer sparse representation (MLSR) model is constructed to improve the recognition rate. Promising results on standard database demonstrate the potential of the proposed approach. To this end, across-database experiments represent a future challenge in facial expression recognition.
